# Authors’ reply

**Published:** 2010

**Authors:** Afekhide E. Omoti, Caroline E. Omoti, Rita O. Momoh

**Affiliations:** Department of Ophthalmology, University of Benin Teaching Hospital, PMB 1111, Benin City, Edo State, Nigeria; 1 Department of Haematology, University of Benin Teaching Hospital, PMB 1111, Benin City, Edo State, Nigeria

Sir,

We thank the authors of this letter for their keen interest on our article. However, we still believe that most of the published work on this topic involves only case reports or small case series.^1^,^2^ The authors have listed a handful of studies that were large series out of the hundreds of publications available on the topic in peer reviewed literature. Of note, is that we did not state that there is a complete lack of studies that included large numbers. We encourage only studies on large sample sizes patients that would accurately reflect issues surrounding this disease. Moreover, we also encourage studies from an alternate viewpoint and agree that they would be of interest to ophthalmologists. Two such studies were conducted by Shields *et al*.^3^ and Demirci *et al*.^4^ however what was reported by them was not the primary focus of our study.

Patients undergoing chemotherapy were excluded because this treatment can cause ocular complications which may cause confusion in deciding whether lesions were due to either lymphoma or treatment.^5^ We concur that it is possible for lymphoma patients to present with features of hemorrhages and vasculitis, but that the more likely presentation would be in the form of confluent, round, or geographic greasy-yellow sub-RPE infiltrates. Such features were not found in any of the subjects of our study cohort. Perhaps there may be racial differences in Black African patients or that the small number of subjects with lymphoma may have affected the outcomes.
